# A Design of Experiments (DoE) Approach to Optimize Cryogel Manufacturing for Tissue Engineering Applications

**DOI:** 10.3390/polym14102026

**Published:** 2022-05-16

**Authors:** Duarte Nuno Carvalho, Cristiana Gonçalves, Joaquim Miguel Oliveira, David S. Williams, Andrew Mearns-Spragg, Rui L. Reis, Tiago H. Silva

**Affiliations:** 1 3B’s Research Group, I3B’s—Research Institute on Biomaterials, Biodegradables and Biomimetics of University of Minho, Headquarters of the European Institute of Excellence on Tissue Engineering and Regenerative Medicine, AvePark, Barco, 4805-017 Guimarães, Portugal; duarte.carvalho@i3bs.uminho.pt (D.N.C.); miguel.oliveira@i3bs.uminho.pt (J.M.O.); rgreis@i3bs.uminho.pt (R.L.R.); tiago.silva@i3bs.uminho.pt (T.H.S.); 2 ICVS/3B’s—P.T. Government Associate Laboratory, Braga, 4805-017 Guimarães, Portugal; 3 Jellagen Limited, Unit G6, Capital Business Park, Parkway, St. Mellons, Cardiff CF3 2PY, UK; david.williams@jellagen.co.uk (D.S.W.); andrew@jellagen.co.uk (A.M.-S.)

**Keywords:** marine biomaterials, marine origin biopolymers, factorial design, optimization, cryo-environment, cartilage tissue

## Abstract

Marine origin polymers represent a sustainable and natural alternative to mammal counterparts regarding the biomedical application due to their similarities with proteins and polysaccharides present in extracellular matrix (ECM) in humans and can reduce the risks associated with zoonosis and overcoming social- and religious-related constraints. In particular, collagen-based biomaterials have been widely explored in tissue engineering scaffolding applications, where cryogels are of particular interest as low temperature avoids protein denaturation. However, little is known about the influence of the parameters regarding their behavior, i.e., how they can influence each other toward improving their physical and chemical properties. Factorial design of experiments (DoE) and response surface methodology (RSM) emerge as tools to overcome these difficulties, which are statistical tools to find the most influential parameter and optimize processes. In this work, we hypothesized that a design of experiments (DoE) model would be able to support the optimization of the collagen-chitosan-fucoidan cryogel manufacturing. Therefore, the parameters temperature (A), collagen concentration (B), and fucoidan concentration (C) were carefully considered to be applied to the Box–Behnken design (three factors and three levels). Data obtained on rheological oscillatory measurements, as well as on the evaluation of antioxidant concentration and adenosine triphosphate (ATP) concentration, showed that fucoidan concentration could significantly influence collagen-chitosan-fucoidan cryogel formation, creating a stable internal polymeric network promoted by ionic crosslinking bonds. Additionally, the effect of temperature significantly contributed to rheological oscillatory properties. Overall, the condition that allowed us to have better results, from an optimization point of view according to the DoE, were the gels produced at −80 °C and composed of 5% of collagen, 3% of chitosan, and 10% fucoidan. Therefore, the proposed DoE model was considered suitable for predicting the best parameter combinations needed to develop these cryogels.

## 1. Introduction

Despite the advances of modern medicine, there is still a severe difficulty in finding adequate donors of tissues and organs to meet the vast patient needs. Due to innumerable limitations, the scientific community has focused more on other alternatives, such as tissue engineering (TE). This approach can create adequate temporary (bio)material scaffolds to promote the regeneration of human tissues without altering the original anatomical and physiological function [1,2]. To perform this demand, (bio)materials should meet some basic requirements such as matching to the native extracellular matrix (ECM), providing a favorable microenvironment to preserve the normal phenotype of cells and their metabolisms; being degradable; and above all, being safe for the patient [3].

In general, these structures can be prepared using a multicombination of two principal approaches: (1) the methodology or procedure to develop the biomaterials such as ionic chemical gelation, (bio)inks for 3D printers, cryo-environments, and freeze-drying, among others [4], and (2) the materials (e.g., polysaccharides, proteins, or polyesthers) and their origin that can be derived from natural resources, such as plant, mammal, and marine sources or synthetic and semi-synthetic materials [5]. In this sense, the methodology, reagents, and bioactive compounds make possible the creation of diverse and unique biomaterial scaffolds with distinct forms, sizes, and properties. 

In the biomaterials group, it is possible to produce different types of scaffolds, such as cryogels that can be obtained from frozen polymeric solutions stimulated by polymerizable precursors that react in temperatures below zero and form an intricated network [6], having as a principal advantage a simple and faster methodology in relation to other procedures. During this process, the interconnected pore network is formed by ice crystals, highly influenced by the freezing temperature, which reflects on time until ice crystal formation: generally, the larger the temperature drop, the faster the freezing time, and smaller the ice crystals [7]. These polymeric solutions can be obtained from natural sources such as the marine environment, from which they can be extracted in a sustainable form, including different bioactive compounds such as collagen, chitosan, and fucoidan [8]. There is a deep concern of the use of materials of mammal origin due to the risks associated with infections (such as bovine spongiform encephalopathy (BSE)), immunogenicity, and rejection for ethical reasons in motivating research on the use of these marine resources [9,10]. Moreover, these marine compounds have been widely reported for their similarities with proteins and polysaccharides (e.g., collagen, hyaluronic acids, and chondroitin sulfates) present in human ECM, supporting a biomimetic approach of biomaterial development mimicking the composition with the native ECM [11].

**Figure 1:**
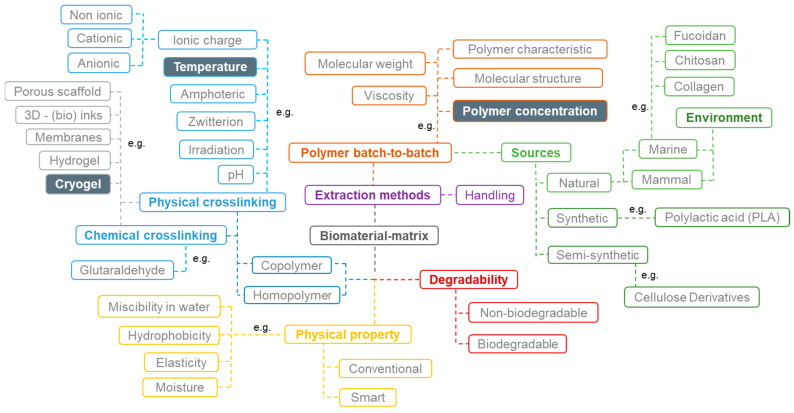
Schematic representation of a direct and indirect variable network. The main variables were: source type (green); polymer batch-to-batch (brown); biomaterial physical properties (yellow); degradability (red); lab experiments and handling (purple); crosslinking type (blue), and biomaterial type (grey).

In the development of biomaterials, each variable (method, origin material, and concentration, among others) can be defined as a different parameter that, individually or grouped, influence the material characteristics, behavior, properties, and consequently, its final application. However, the interaction between different parameters is complex since it depends not only on direct variables but also on indirect multivariable, such as surrounding temperature, moisture variations, and handling procedures [12], as shown in Figure 1. In the case of cryogels, the temperature (below zero), the chosen sources, the initial polymeric concentration, and their basic structural properties (e.g., the type of ionic charge available) are crucial for forming these types of biomaterials [13]. The selection of the most relevant parameters and the values promising improved performance should be made with caution, envisaging the final application or approach. Therefore, it is crucial to prudently reflect on the parameters to be considered, and for instance, construct a schematic representation of their role and interactions that may affect the final result.

At the moment, much research has been reported on the impact of specific parameters (i.e., the individual effect of each factor), such as the temperature [14] or pH [15], on the development of cryogels. However, little is known about the interactions of the chosen parameters with the biomaterial behavior, i.e., how they can influence each other toward improving their physical and chemical properties, given that it is difficult to instinctively recognize and predict the interactions between several factors. To achieve this quest, factorial design of experiments (DoE) and response surface methodology (RSM) are known as statistical tools to identify the most influential parameter and optimize production processes [16,17]. In brief, this method usually uses linear regression and analysis of variance (ANOVA) mathematical models to extrapolate and predict the interaction of the parameters, their interrelationship, and the optimal point [18]. DoE and RSM have been extensively applied in many areas, which includes the optimization of heterologous protein expression [19], bioactive extraction methods [20,21], and scaffold production, namely, electrospun materials [22], membranes [23], and hydrogels [24], among others. However, to the best of our knowledge, there are no reports in the literature on the optimization of biomaterial systems based on cryo-environments envisaging biomedical approaches.

In summary, in this work, the DoE method was applied to find the optimal values for pre-defined parameters (1) temperature, (2) collagen concentration, and (3) fucoidan concentration) which significantly influence the physico-chemical properties of cryogels developed with jellyfish collagen, chitosan, and fucoidan, and their behavior in contact with cells, being decisive for tissue engineering purposes. A three-level full factorial design for three factors (*n* = 27), Box–Behnken design, was applied. This resulted in a large dataset that reveals the importance of statistical parameter studies for a better understanding of the biomaterial behavior and process optimization.

## 2. Materials and Methods

### 2.1. Materials

Collagen from Jellyfish *Rhizostoma pulmo* (jCOL) was provided by Jellagen Pty Ltd. (Cardiff, UK). Fucoidan obtained from brown algae *Fucus vesiculosus* (aFUC) was supplied by Marinova (Maritech Fucoidan, FVF2011527 Marinova, Australia). In addition, chitosan was extracted from squid pens obtained from giant squids *Dosidicus gigas* (sCHT), with a deacetylation degree (DD) of 81.8%, and a molecular weight (M_w_) of 334 kDa. According to the PCT patent WO/2019/064231 [25], the raw material was converted into chitosan using a deproteinization and deacetylation method with one single alkaline process under a nitrogen (N_2_) atmosphere at 75 °C for 2 h.

### 2.2. Marine Cryogel Preparation

Firstly, the collagen and chitosan were dissolved in ammonium acetate (0.15 M NH_4_OAc/0.2 M AcOH) (pH 4.5). Then, fucoidan was dissolved in a different container with ultrapure water (Milli-Q). Table 1 lists the initial solution concentration of each polymer and respective cryogel formulations. Finally, the marine solutions were gently mixed using an Ultra-Turrax^®^ T18 overhead Blended, IKA Works Inc., Wilmington, NC, USA) in lower rotations to create a homogenous solution (and to avoid bubbles). 

**Figure 2:**
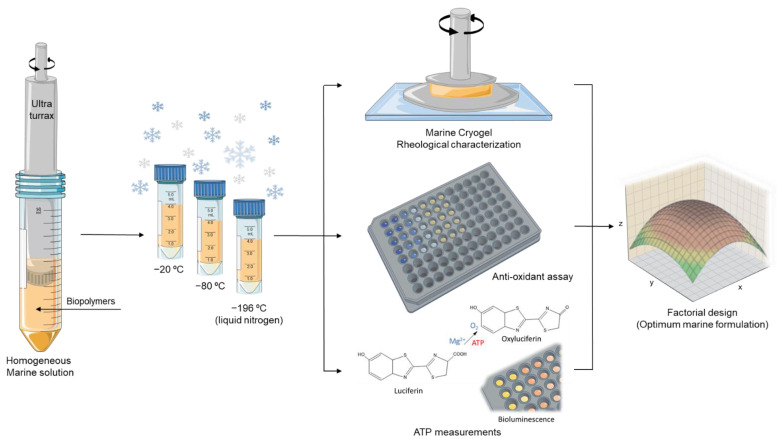
Schematic representation of the marine cryogel formation procedure comprising homogenization and freezing at different temperatures and then its characterization, aiming for a factorial design to extrapolate the optimum marine cryogel formulation according to the obtained results from rheology, anti-oxidants activity, and ATP measurements.

To form the cryogels, the natural crosslinking between the biopolymers (by electrostatic interactions) was performed in a cryo-environment process, where the mixed solutions were placed overnight in temperatures below zero (−20 °C, −80 °C, and −196 °C) and then transferred for a few hours to the fridge (4 °C). To neutralize the pH, the cryogels were placed several times into a D-MEM solution (cell culture medium) with the intention of not compromising the viability of the cells by the presence of acidic material. The entire cryogel production procedure is demonstrated in Figure 2.

### 2.3. Rheological Measurements

The rheological oscillatory properties were assessed using a Kinexus pro+ rheometer and rSpace software (Malvern Instruments, Worcestershire, UK) for the data acquisition. The equipment has a top measurement geometry (8 mm in diameter) and a bottom plate pedestal, both with stainless steel (316 grade). The experiments were performed to investigate the cryogel viscoelastic properties through their mechanical spectra (frequency sweep curves) using a range of 0.1 Hz to 10 Hz at 25 °C, with the value of strain (1%) obtained from the Linear viscoelastic region (LVER). The LVER strain value was firstly determined through a strain sweep test (0.01–10%) using a constant frequency (1 Hz) at room temperature (25 °C). The oscillation experiments could be performed within this linear range without damaging the sample structure. 

Additionally, some structural parameters, such as the average mesh size (*ξ*/nm) and the crosslinking density (*n_e_*/(mol/m^3^)) of the cryogels, could be calculated using the data obtained from the rheological oscillatory experiments [26]. Regarding this, *ξ*/nm was defined as the distance between the crosslinking points that can be established by the rubber elastic theory (RET), Equation (1):

(1) $$\xi /\mathrm{nm}\;=\sqrt[{3}]{\frac{RT}{{G^{\prime }\;N}_{A}}}\times {\;10}^{9}$$

where G′ is the storage modulus, N_A_ is the Avogadro constant (6.022 × 10^23^), *R* is a value of gas constant (8.314 J/K mol), and *T* is the temperature in Kelvin (25 °C = 298.15 K) [27]. The values obtained with these units were in meters and then converted to nanometers.

The *n_e_*/(mol/m^3^) is nominated by the number of elastically active connection points in the network per unit of volume, calculated by RET, Equation (2):

(2) $$n_{e}\;=\;\frac{G_{e}}{RT}$$

where G_e_ is the plateau value of storage modulus measured by the frequency sweep test [28].

### 2.4. Antioxidant Assay

Antioxidant analysis was performed using a straightforward method to measure the amounts of phenolic compounds [29]. The quantification of total phenolic contents was performed according to the traditional Folin–Ciocalteu reactive methodology, a well-known method (Folin–Ciocalteu index) [30,31]. This reaction involves oxidation, in an alkaline solution, of phenols by the yellow Folin–Ciocalteu reagent (a mixture of phosphomolybdate (H_3_PMo_12_O_40_) and phosphotungstate (H_3_PW_12_O_40_)), and the colorimetric measurement of the resultant combination of blue oxides, molybdenum, and tungsten, is proportional to the total phenolic compounds. In brief, 1 mg/mL of each marine cryogel condition was mixed with deionized (DI) water, the Folin–Ciocalteu reagent, and 15% sodium carbonate (Na_2_CO_3_). The microplate with the final mixtures was immediately placed in a 50 °C oven and removed after 10 min. After establishing at room temperature, the absorbance was read at 740 nm using a microplate reader (Synergy HT, Bio-Tek Instruments, Winooski, VT, USA). Caffeic acid was used as a standard to establish the calibration curve.

### 2.5. ATP Measurements

Intracellular adenosine triphosphate (ATP) levels were quantified to indicate viability and possible cell proliferation. CellTiter-Glo luminescent assay (Promega, Madison, WI, USA) was used for this analysis [32]. Firstly, the chondrocyte-like cell lines (ATDC5) were cultured on top of each marine cryogel inside a 96-well plate (3 × 10^4^ cells/well) with Dulbecco’s modified Eagle’s medium-low glucose (DMEM, Sigma-Aldrich, Burlington, MA, USA) supplemented with 10% fetal bovine serum (Alfagene, Waltham, MA, USA) and 1% antibiotic–antimycotic solution (Gibco, Cambridge, UK) for 24 h. After that, at each time point (24, 48, and 72 h), the reagent CellTiter-Glo was added in a 1:1 ratio (e.g., add 100 µL reagent to 100 µL of medium-containing cells) and incubated at room temperature for 30 min to allow the cellular lysis. The phosphorescence luminescence at 450–600 nm was then read using a fluorescence spectrometer with the capacity to execute phosphorescence experiments (JASCO FP-8500, Hachioji, Tokyo, Japan).

### 2.6. Statistics

Data analysis was performed using the OriginLab Pro 2019b program to analyze the rheological oscillatory behavior. Complementary statistical analysis of rheology, antioxidant activity (by phenolic groups), and ATP quantification results were performed by ANOVA followed by Tukey’s post hoc test, using GraphPad Prism 8.0.1 (GraphPad Software, Inc., La Jolla, CA, USA). Differences between the groups with a confidence level of 95% (*p* < 0.05) were considered statistically significant. All results are presented as mean ± standard deviation.

Response surface methodology (RSM) is an empirical statistical modeling procedure used for multiple regression analysis applying quantitative data acquired from adequately designed experiments to solve multivariate equations simultaneously [33]. A 3^3^ Box–Benkhen experimental design was applied in this research to optimize hydrogel formulation and study the influence of 3 parameters with 3 levels on the structure’s performance, resulting in 27 experiments. Each parameter, an independent variable, was coded at 3 levels between −1 (low level), 0 (middle point), and +1 (high level). Coding of the variables was achieved by using Equation (3) [33]:

(3) $$x_{i}=\frac{X_{i}-X_{cp}}{\Delta X_{i}},\;\;\;\;\;i=1,2,3,4,\ldots k$$

For an independent value, *x*_i_ is its dimensionless value; *X*_i_ its actual value; and *X*_cp_ is its real value at the center point. Moreover, Δ*X*_i_ is the step change of the actual value corresponding to a unit variation for the dimensionless value of the variable i.

The carefully considered parameters were temperature (*x*_1_), collagen concentration (*x*_2_), and fucoidan concentration (*x*_3_) (Table 2). The structure’s performance was measured by their rheological behavior, antioxidant potential, and cellular behavior (ATP quantification). Table 2 lists the process parameters (factors *x*_1_, *x*_2_, and *x*_3_) and levels for hydrogel fabrication, with the coded (−1, 0, and 1) and actual values. 

The design included the dependent variables (G′/Pa and Antiox/(µg/mL^−1^), and ATP/RLU) was solved separately and used to find the optimal conditions by fitting a polynomial model, which gave the response as a function of relevant variables using RSM (Statistica, 12, Stat-Ease Inc., Minneapolis, MN, USA, 2014). The regression model of the present experimental Box–Behnken design system is described by Equation (4), to evaluate the effect of each independent factor on the response [34].

(4) $$Y=\beta_{0}+\beta_{1}x_{1}+\beta_{2}x_{2}+\beta_{3}x_{3}+\beta_{12}x_{1}x_{2}+\beta_{13}x_{1}x_{3}+\beta_{23}x_{2}x_{3}+\beta_{11}x_{1}^{2}+\beta_{22}x_{2}^{2}+\beta_{33}x_{3}^{2}$$

*Y* corresponds to the predicted response; for instance, for the G′/Pa; *x*_1_, *x*_2_, and *x*_3_ are the coded levels of the independent factors (temperature and concentration of collagen and fucoidan). The coefficients of the regression are *β*_0_ for the intercept term; *β*_1_, *β*_2_, and *β*_3_ the linear coefficients; *β*_12_, *β*_13_, and *β*_23_ the interaction coefficients; and *β*_11_, *β*_22_, and *β*_33_ the quadratic coefficients.

## 3. Results and Discussion

### 3.1. Rheology Oscillatory Behavior

**Figure 3:**
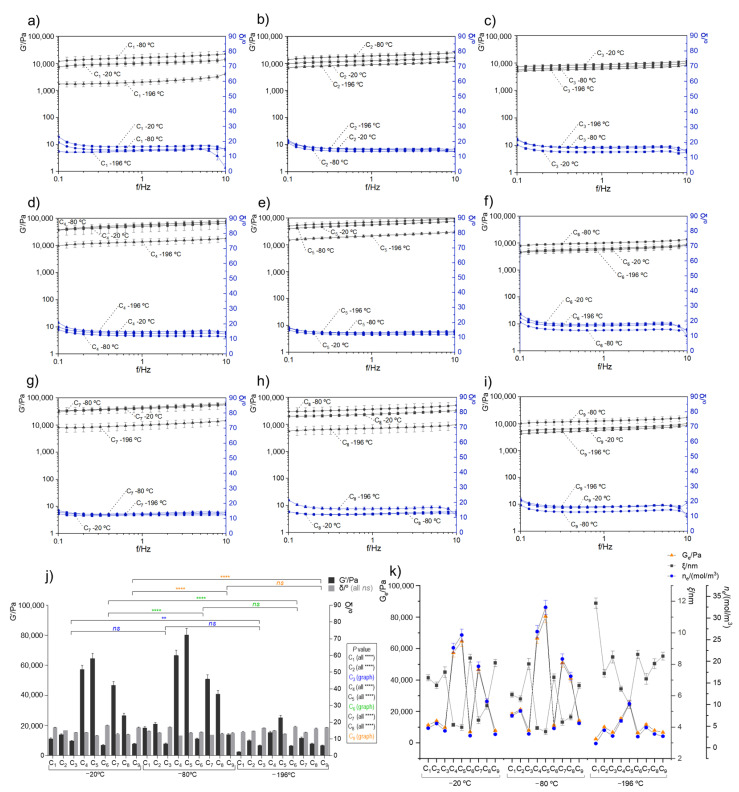
Oscillatory rheological behavior of different marine origin cryogels (**a**–**i**) corresponded to the developed cryogels C_1_ to C_9_, respectively, produced at −20, −80, and −196 °C. Data show the mean of three values from independent experiments and the standard error for G′/Pa (black lines) and the phase angle δ/° (blue lines). (**j**) Comparison of each marine cryogel as a function of G′/Pa and the δ/°, with the average stable zone between 0.5 to 5 Hz. All differences between samples were statistically significant **** *p* < 0.0001 except those represented with the symbols of ** (*p* < 0.01) and *ns* (not significant). (**k**) Comparative analysis of the storage modulus at the plateau (G_e_/Pa), mesh size (*ξ*/nm), and the crosslinking density (*n_e_*/mol/m^3^) of the developed marine cryogels.

The rheological oscillatory experiments in distinct marine cryogel compositions were performed to understand their mechanical properties. This analysis consisted of measuring the elasticity modulus (G′), the viscosity modulus (G″), and the phase angle (δ/°) obtained from G″/G′ (or damping factor, tan δ) to evaluate the resistance to shear stress within a frequency range and performed at 25 °C. The obtained results and the respective statistical analysis are shown in Figure 3, with G′ and δ being barely independent of frequency (between 0.1 and 10 Hz) for all the analyzed cryogels. Moreover, the composition of the cryogels influenced the rheological behavior, as illustrated by the variability of G′ shown in Figure 3j).

When G′ > G″ and the tan δ < 1, the sample behavior tended to have a solid gel character (strong gel), which reflects the connectivity of the polymeric network [35,36]. The obtained oscillatory rheological experiments for the different cryogels (Figure 3a–i) exhibited exactly this behavior (viscoelastic character), thus revealing the presence of physical interactions between the polymers and resulting in cohesive matrices [26]. It is possible to quantify how strong these bonds are by determination of the distance between crosslink (i.e., average of mesh size) (*ξ*/nm) and the crosslinking density (*n_e_*/(mol/m^3^)) using the well-known rubber elastic theory (RET) and some rheological parameters such as the storage modulus (G′) value that can also be designated as G_e_ [27,37].

According to G_e_ results obtained at the plateau between 1–10 *f*/Hz, it is clear that the cryo-environment temperatures and the ratio of the polymers directly influenced the rheological properties of the biomaterials due to differences in the internal network. Figure 3j shows an overview of all tested biomaterials and reveals that the temperature that provided a higher viscoelastic character was −80 °C, followed by −20 °C. In comparison, the formulations submitted to liquid nitrogen (−196 °C) resulted in lower internal network formation. This lower bond formation capacity may be related to the fast-freezing environment of the polymer solution, quickly hampering polymer mobility, which does not provide enough time for the material to organize properly, namely, to interact with the other biopolymers (through electrostatic attraction, among others), resulting in a less cohesive structure. Actually, the internal polymeric network structure in each formulation is promoted between the positively charged groups present on collagen and chitosan samples (i.e., protonated amines) and the negatively charged groups present on fucoidan (i.e., ester sulfates and eventually, carboxylate groups) [38]. In this regard, a cohesive structure is not only promoted by the appropriate cryo-environment procedure but also highly depends on greater or lesser availability of the positive and negative charges that are present in each formulation. Within each processing temperature, the formulations that demonstrated better rheological properties were C_5_ and C_4_, followed by C_7_ and C_8_. This phenomenon was mostly observed in formulations that contained higher collagen concentrations (i.e., C_5_, C_7_, and C_8_) when calculating the total polymer mass in each cryogel formation. Fucoidan also has a vital function on biomaterial formation through electrostatic interactions, dependent on fucoidan concentration [39]. However, our rheological results demonstrated that despite the relevance of the presence of fucoidan, an extra concentration could respond negatively in the formation of bonds, which consequently would be reflected in rheological properties [40,41]. Thus, a balance between collagen and fucoidan amounts is required to create structures with significant stability. 

Figure 3k shows a correlation between the G_e_ values with the calculated *ξ* and *n_e_*/(mol/m^3^). In general, the cryogels with better rheological properties (i.e., C_5_ and C_4_ at −80 and −20 °C) had the lowest mesh size (varied from 3.71 ± 0.17 nm to 4.44 ± 0.21 nm) and a crosslinking density that were proximal to the G_e_ values (followed the same trend), which confirmed excellent stability on these formulations compared to other samples, in which *ξ* varied from 5.79 ± 0.18 nm to 8.46 ± 0.37 nm. This behavior is in accordance with the literature since it is expected to exist proportionally between the rheological parameters and the stiffness behavior of the biomaterials.

### 3.2. Antioxidant Activities

Marine sources have been widely studied for their biological properties, attributed to different compounds and metabolites that are produced to protect the living being when exposed to harsh environmental conditions, such as abiotic and biotic factors, light intensity, salinity, and ultraviolet radiation, among others [42]. In the case of brown seaweeds, there are two major bioactive components—sulfated polysaccharides (SPs), namely, fucoidan, and polyphenol compounds—that have been classified as having a significant antioxidant activity [43]. Some authors defend the importance of the presence of phenolic compounds in commercial fucoidan samples due to the fact that the phenols are the main contributors to the antioxidant activities compared with fucoidan, which together can increase the biological properties without bringing negative consequences [44]. In particular, it was reported by Murray et al. [45] that the extracts of *Fucus vesiculosus* commercialized by Marinova (Australia), used in the present study, can contain up to 28% of polyphenols.

The Folin–Ciocalteu (F–C) method was used as a straightforward method to compare the samples regarding the polyphenolic antioxidant activity, also being indirectly interrelated with the amounts of fucoidan present on cryogel samples. This approach is equivalent to the work by Palanisamy et al. [46], where they concluded that the antioxidant activity can be correlated with the concentration of fucoidan. Briefly, this method is based on a reaction of electron transfer that involves a mixture of phosphomolybdate and phosphotungstate to determine the antioxidant activity. Thus, this methodology measures the reductive capacity of the antioxidants, being widely applied to determine these contents in plant-derived food, as well as in other biological samples, such as fucoidan [42,47]. Other antioxidant assays could have been used, such as DPPH (2,2-diphenyl-1-picryl-hydrazyl-hydrate), ORAC (oxygen radical absorbance capacity), and ABTS (2,2′-Azinobis-(3-Ethylbenzthiazolin-6-Sulfonic Acid), but the outcome would be the total antioxidant capacity, and it would not be possible to distinguish between the activities promoted by the polyphenols and fucoidan [48,49].

**Figure 4:**
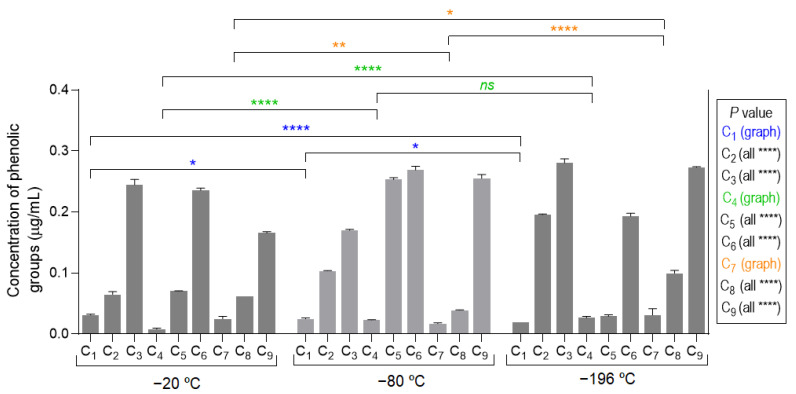
Assessment of antioxidant activity of marine origin cryogels (C_1_ to C_9_, produced at −20, −80, and −196 °C) by the quantification of phenolic groups. All samples (comparing the different temperatures) showed statistical significance of **** *p* < 0.0001 except those represented with the symbols of * (*p* < 0.05), ** (*p* < 0.01), and *ns* (not significant).

The obtained results of the total concentration of phenolic groups on the developed marine cryogels, depicted in Figure 4, showed a growth trend within each group of three samples (i.e., C_1_ to C_3_, C_4_ to C_6_, and C_7_ to C_9_), with similar values between those groups. These results were in good agreement with amount of fucoidan in the biomaterial formulation (Table 1). The compositions that contained a lower concentration of fucoidan (i.e., C_1_, C_4_, and C_7_) showed lower antioxidant activity, while those that had a higher concentration (i.e., C_3_, C_6_, and C_9_) had, in the same way, higher antioxidant functional activity.

### 3.3. ATP Activity

**Figure 5:**
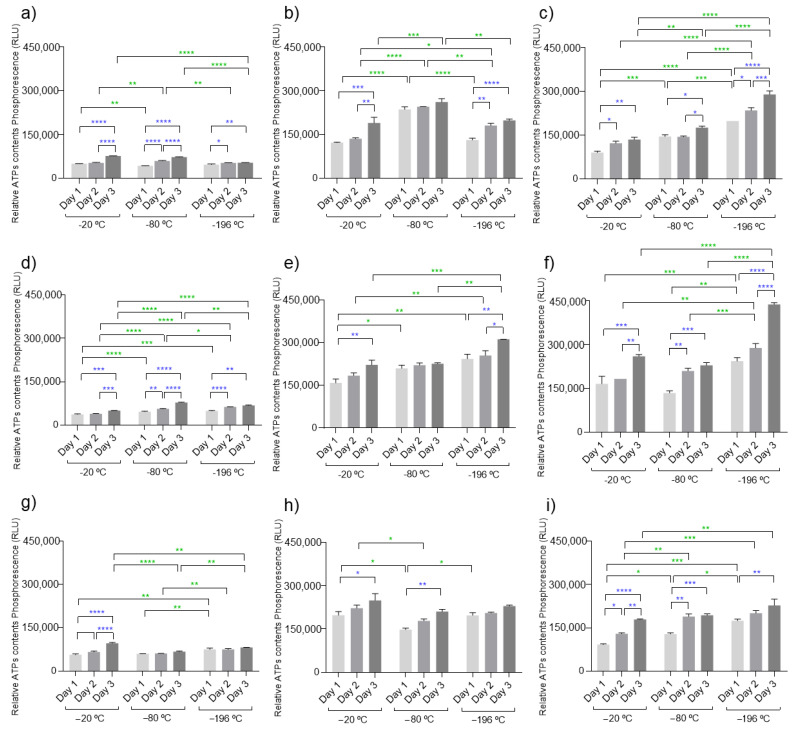
ATP cell measurements using ATDC5 cells in direct contact with all developed marine cryogels C_1_ to C_9_ ((**a**–**i**), respectively) at −20, −80, and −196 °C. There was a direct relationship between the measured phosphorescence and the concentration of ATP. The samples showed statistical significance of * (*p* < 0.05), ** (*p* < 0.01), *** (*p* < 0.001), **** *p* < 0.0001, or were not significant (not represented). The symbols of statistical analysis noticeable by blue color correspond to the comparison between each condition in relation to the time points while the symbols expressed by green color represent the comparison between the different temperatures of each marine condition.

Adenosine triphosphate (ATP), also called molecular unity of energy currency, is an intracellular nucleotide that provides energy for many metabolic processes in every living cell and is produced by ATP synthase [50]. Nowadays, ATPs can be measured using a (bio)luminescence or phosphorescence assay (similar emission light) [51]. During the chemical reaction, the enzyme luciferase has the ability to release energy in the form of light (luminescence) on the presence of its substrate luciferin, Mg^2+^ and ATP [52]. It is described in the literature that there exists a linear relationship between the amount of ATP present in samples and the amount of light produced, which is a direct indicator of cellular metabolic activity [53]. The amounts of ATP present in cells cultured on the developed marine cryogels are shown in Figure 5.

The amounts of ATP were quantified on chondrocyte-like cells (ATDC5) cultured in direct contact with the developed cryogels during three time points (24, 48, and 72 h) as a measure of cellular metabolic activity. In general, the results demonstrated that these marine biomaterials do not compromise the metabolic activity of cells, since during the experiments, an increase in ATP content was observed, which is a good indicator of cell viability when encapsulated on the biomaterial. Typically, in favorable conditions, the energy expenditures by cells during the first hours are dedicated especially to cell adhesion, while the rest of the time is devoted to normal cell functionalities such as cell proliferation. Cell adhesion is essential for survival and communication (cell-to-cell) since it allows stimulation of important signals for cell migration, the cell cycle, proliferation, and taking the example of stem cells, for their differentiation [54]. Our data also showed that these cell types have a preference for biomaterials with a higher concentration of polymers on their formulations. This behavior was observed when comparing to the formulations C_1_, C_4_, and C_7_ (lower concentration of fucoidan) to the other formulations, and showed the same tendency in all temperatures used. The concentration of collagen in the biomaterial did not show a significant influence on ATP quantification, suggesting that the presence of fucoidan was more impactful for the short time that the cultures were evaluated. The processing temperature did not significantly influence the cellular behavior, as no pattern could be identified on the variation in ATP content from cells cultured in each biomaterial formulation processed at different temperatures. This was certainly influenced by the short time that the cell cultures were studied, as hydrogels with a more compacted structure (as the hydrogels resulting from biomaterials processed with liquid nitrogen) are expected to hamper cell proliferation, and experiments with more extended culture periods might show some temperature influence. Nevertheless, under the studied conditions, the amount of fucoidan in biomaterial composition seems to be the determining factor for the cellular outcome. 

## 4. Factorial Design

Box–Behnken designs (BBD) are a practical response surface design (RSD), such as the central composite design, that provides data based on experiment variables and overall experimental error. These designs possess excellent symmetry and rotatability, with minimal experimental runs [55]. The systematic optimization of the structure’s performance was performed with the help of the BBD matrix, indicating 27 experimental trials, and the response data for characterization of those structures are represented in Table 3. This table was obtained primarily to perform the experiment concerning the considered factors and levels. Then, after performing the 27 experiments required, it was completed with the response data to analyze the response surface design.

**Figure 6:**
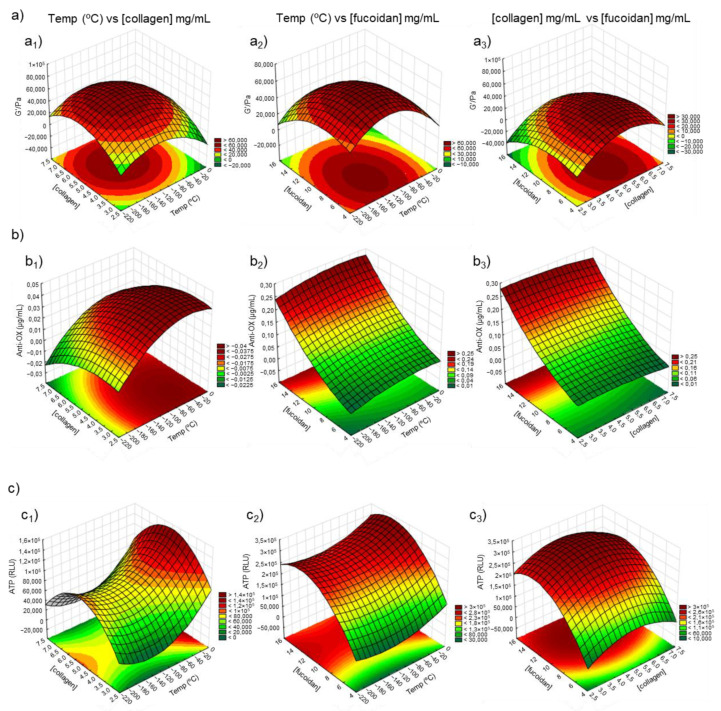
Factorial surface response of (**a**) rheological data (G′/Pa), (**b**) antioxidant activity (µg/mL), and (**c**) ATP (RLU) measurements on marine origin cryogels. The images of (**a_1_**, **b_1_**, and **c_1_**) demonstrate the correlation between the temperature (°C) vs. concentration of collagen ([collagen]); (**a_2_**, **b_2_**, and **c_2_**) represent the correlation between the temperature in relation to the concentration of fucoidan ([fucoidan]) while the images of (**a_3_**, **b_3_**, and **c_3_**) show the correlation between the concentration of collagen vs. the concentration of fucoidan.

The response surface analysis was performed for all the response variables based on the selected model. The statistical and correlation analysis of the model’s response was performed with 3D response surface plots, histogram of distribution, Pareto plots, and analysis of variance (ANOVA). Figure 6 illustrates the 3D response surface plots for the studied responses. To draw the 3D surfaces, the ATPs (RLU) chosen were those from the 72 h, since this 3rd timepoint is when the normal metabolic function of the cells occurs (cellular proliferation), while during the 1st or 2nd timepoints, the cells are using energy for cell adhesion in the polymeric structure.

Nine response surface plots were generated to exhibit the effect of the three factors on the responses: G′ (Pa) (Figure 6a), antioxidant concentration (µg/mL) (Figure 6b), and ATPs (RLU) (Figure 6c). The quadratic models showed different forms for each response surface: “mound-shaped” maximum (a_1_, a_2_, a_3_, b_1_, and c_3_), saddle point (c_1_ and c_2_), and rising ridge (b_2_ and b_3_) [56], with the optimum point able to be effortlessly observed on the first type since the region of optimum response was located inside the experimental region. Concerning the rheological behavior (Figure 6a), the optimum value was predicted to be inside the experimental region in the 3 surfaces, the optimum value (G′ of 6.47 × 10^4^ Pa) being indicated for a temperature of −126 °C and a concentration of collagen and fucoidan of 5.3 mg/mL and 7.7 mg/mL (Table 4). Unfortunately, the optimum temperature could not be implemented in the laboratory environment, limited to −80 or −196 °C (liquid nitrogen) due to equipment limitations. However, according to the DoE results, the formulation that mostly resembled these values was the formulation C_5_ at −80 °C, which comprised the percentage (*w*/*v*) of each polymer in the original solution, i.e., 5% collagen, 3% chitosan, and 10% fucoidan followed by C_4_ at −80 °C. These results are consistent with our previous work [8], since we also found a trend for the formulation that comprised a similar polymeric concentration. Regarding the results obtained for the concentration of the antioxidants (µg/mL), the optimum value was not completely inside the experimental region surface. Thus, an optimum value (0.13 µg/mL) was predicted for a temperature of −54 °C, and 3.5 mg/mL of collagen concentration. No optimum value of fucoidan concentration for antioxidant concentration was found since the surfaces with the independent variable were from the type rising ridge [56]. Finally, the independent variable of ATP had the optimum value (ATP of 2.56 × 10^5^ RLU) indicated for a temperature of −145 °C and a concentration of collagen and fucoidan of 5.1 mg/mL and 12.5 mg/mL, respectively (Table 4). It was impossible to achieve a formulation exhibiting the best values for all the properties being assessed (rheological behavior, antioxidant activity, and ATP quantification) and compromises or priorities needed to be established, depending on the final application. In any case, according to the obtained analysis, the formulations that mostly resembled the obtained DoE values were the samples C_5_ and C_6_ at −196 and −80 °C.

At this point, it is essential to mention that when working with natural materials, many mathematical tools and models fail or are not completed, which is probably why they are rarely applied. This happens for numerous known reasons, such as reproducibility problems, the impossibility of repeating an optimal point, or the high material sensitivity. Contrary to what happens in other areas of more exact science where a wide range of options are available, even in this situation, limitations regarding available equipment can be an obstacle. Still, applying these tools is an enabling option to redirect the research work to better performing conditions, even when the experimental conditions do not allow one to achieve the final purpose of obtaining a full and irrefutable optimum point.

The experimental design also enabled us to build for each group of response data: a histogram of distribution, Pareto plot, and the correlation between what was observed and the predicted values (Figure 6). The histogram of distribution, showing frequency distributions, assesses normality (bell-shaped and symmetric about the mean). The Pareto chart shows a t-statistical test for each effect, where each bar represents the standardized effect, compared to a t-critical value (vertical line). Thus, it renders a method that detects the factor and interaction effects that are most important to the process or design optimization [57]. A plot of observed values (measurements) and predicted values (DoE regression) was also drawn. The blue diagonal line marks the best-case scenario where measurement and prediction ideally resemble each other [57].

**Figure 7:**
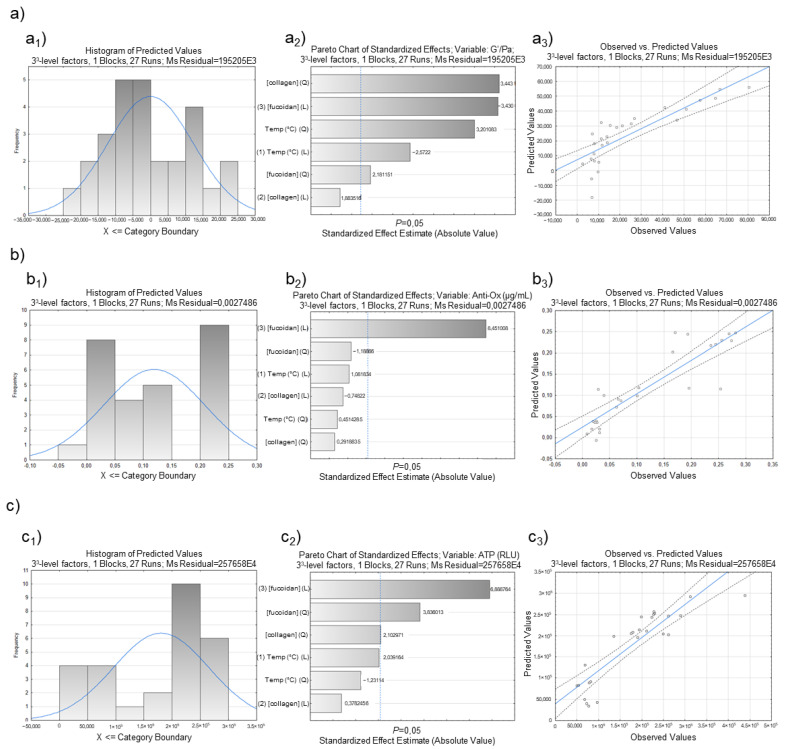
Factorial response of (**a**) rheological data (G′/Pa), (**b**) antioxidant activity (µg/mL), and (**c**) ATP (RLU) measurements on marine origin cryogels. The images (**a_1_**, **b_1_**, and **c_1_**) demonstrate the histogram of distribution; (**a_2_**, **b_2_**, and **c_2_**) represent the Pareto charts of standardized effects of the factorial design, while the images of (**a_3_**, **b_3_**, and **c_3_**) show the Box–Behnken design plot for predicted versus actual values for each response variable.

A better quality of the experimental design was perceived for the data with rheological properties (Figure 7a) when observing the reached plots globally. The histogram of distribution for those data (Figure 7a1) showed a distribution near normality, while the others were slightly distant from it (Figure 7b1,c1). The Pareto chart also displays the statistically relevant effect of each factor on the response, and it is a helpful method to observe the results. The dotted line on the Pareto charts represents the t-critical value, and the effects on its right are significant [58]. Given the proximity of all variables, it is possible to state that the studied independent variables were significant to the process. The concentration of fucoidan followed by the concentration of collagen were the most significant. The correlation between what was observed and the predicted values makes it possible to state that the DoE regression function provided an acceptable approximation to reality in the investigated area. The regression was used to find the predictive equation, i.e., the quadratic model in coded units, for rheological data (Equation (5)), antioxidant activity (Equation (6)), and ATPs (Equation (7)).

(5) $$Y=-14.6\times 10^{3}-0.7\times 10^{3}x_{1}+52.2\times 10^{3}x_{2}+7.7\times 10^{3}x_{3}-3x_{1}^{2}-4.9\times 10^{3}x_{2}^{2}-0.5\times 10^{3}\beta_{22}x_{3}^{2}$$

(6) $$Y=-10.6\times 10^{-3}-0.1\times 10^{-3}x_{1}+11\times 10^{-3}x_{2}+0.5\times 10^{-3}x_{3}-1\times 10^{-6}x_{1}^{2}-1.6\times 10^{-3}x_{2}^{2}+1\times 10^{-3}x_{3}^{2}$$

(7) $$Y=-0.4\times 10^{6}+1.1\times 10^{3}\;x_{1}+80.1\times 10^{3}x_{2}+0.1\times 10^{6}x_{3}+4x_{1}^{2}-10.9\times 10^{3}x_{3}^{2}-3.2\times 10^{3}x_{2}^{2}$$

where, *Y* corresponds to the predicted response, and *x*_1_, *x*_2_, and *x*_3_ are the coded levels of the independent factors (temperature and concentration of collagen and fucoidan).

The predictive equation, describing the polynomial model for the experimental response, was validated by analysis of variance (ANOVA) as shown in Table 5.

According to the ANOVA results (Table 5), the quadratic model, including linear interactions, fitted acceptably to the experimental data giving coefficients of determination (R^2^) ranging from 0.69 to 0.78. The coefficient of determination (R^2^) indicates if the predictive equations adequately describe the experimental values [59]. Globally, the obtained R^2^ values were not as close to one as desired. Nevertheless, natural products usually have such an impact on results. Additionally, their use regularly leads to difficulties of reproducibility (batch-to-batch variability) [60], processing, homogeneity, and the biological complexity of the molecules.

The regression coefficients and the interaction between independent factors can be considered statistically significant for *p*-values lower than 0.05, with a 95% confidence interval [61]. Thus, according to the ANOVA results (Table 5) and the Pareto chart (Figure 7a_2_,b_2,_c_2_), fucoidan concentration’s linear and quadratic terms were the factors that most affected the performance of the structures. Furthermore, as expected in the rheological properties, the temperature factor was more critical than in the other two parameters chosen due to how temperature can directly influence the internal structural network of the biomaterials, making them more cohesive or weaker. The rheological properties are usually temperature-dependent for these kinds of samples [62]. 

In this way, the best conditions regarding the rheological properties were C_5_ −80 °C followed by C_4_ −80 °C, whereas concerning the antioxidant activity, they were C_2_ −80 °C and C_3_ −80 °C, and in the quantification of ATPs, they were C_5_ −80 °C, C_6_ −80 °C, C_5_ −196 °C, and C_6_ −196 °C. Globally, the most recurred formulation was C_5_ at a temperature of −80 °C, being herein considered as the best formulation among the ones addressed. However, as mentioned above, it must be stressed that it is impossible to determine a formulation exhibiting the best values for all the assessed parameters, and choices must be made. In that case, a validation of the determined conditions to obtain the best of each of the parameters should be made, which would lead to an optimization process with respect to the selected parameter. [59]. As abovementioned, it was not possible to do those validations particularly due to limitations on freezing equipment (only determined values were possible, which did not include the value determined as the optimum to maximize the value of each of the different parameters), but that was not the goal of the present work. Being aware of the practical limitations of the application of the model to this experiment, we aimed to present it as a useful tool to give us a correct direction regarding the conditions that most influence each parameter toward the design of better-performing formulations.

## 5. Conclusions

The factorial design of experiments (DoE) model is an effective statistical experimental tool for identifying the most significant parameter(s) and demonstrating each parameter’s effects according to their interactions. The present experimental work focused on identifying the better-performing biomaterial according to a carefully chosen set of parameters, such as the concentration of the polymers (collagen and fucoidan) and the cryo-environment temperature, which promoted the ionic crosslinking between the polymer-charged groups on the cryogels. Data on rheological oscillatory measurements, antioxidant activity, and adenosine triphosphate (ATP) concentration as a measure of cell metabolic activity showed that fucoidan concentration was the parameter that most significantly influenced cryogel formation, while the temperature was the variable that most influenced the rheological properties. The biomaterial that better fit the conditions determined by the DoE was the formulation C_5_ at −80 °C, which comprised the percentage (*w*/*v*) of each polymer in the original solution, i.e., 5% collagen, 3% chitosan, and 10% fucoidan. Moreover, depending on the final approach, the DoE provided a valuable indication of expected behavior of certain formulations within the limits of the model, avoiding unnecessary and excessive characterizations and waste of products, thus being a valuable tool for improved biomaterial design. 

## Figures and Tables

**Table 1:** Cryogel composition prepared by blending three marine origin biopolymers using different temperatures below zero to promote the gelation (ratio of each biopolymer in original solution and their distribution percentage after biomaterial formation).

Hydrogel Abbreviationratio 1:1:1 (100%)	% of Polymer in the Original Solution			Freezing Temperature
	Collagen	Chitosan	Fucoidan	
**C_1_** (T °C)	3; (27.27)	3; (27.27)	5; (45.46)	−20 °C −80 °C −196 °C (liquid nitrogen)
**C_2_** (T °C)	3; (18.75)	3; (18.75)	10; (62.5)	
**C_3_** (T °C)	3; (14.29)	3; (14.29)	15; (71.42)	
**C_4_** (T °C)	5; (38.46)	3; (23.08)	5; (38.46)	
**C_5_** (T °C)	5; (27.78)	3; (16.67)	10; (55.55)	
**C_6_** (T °C)	5; (21.74)	3; (13.04)	15; (65.22)	
**C_7_** (T °C)	7; (46.67)	3; (20)	5; (33.33)	
**C_8_** (T °C)	7; (35)	3; (15)	10; (50)	
**C_9_** (T °C)	7; (28)	3; (12)	15; (60)	

% *w*/*v* of polymer in the original solution; (% *w*/*w* of the total polymer mass in each cryogel formation). (T °C) is the respective freezing temperature.

**Table 2:** Coded levels and the variables of the 3^3^ Box–Behnken factorial design.

Variable	Factors	Level		
	*x*	−1	0	1
**Temperature (°C)**	*x*_1_	−20	−80	−196
**[collagen] (% *w/v*)**	*x*_2_	3	5	7
**[fucoidan] (% *w/v*)**	*x*_3_	5	10	15

**Table 3:** Experimental runs for Box–Behnken with 3^3^ factorial design. Coded levels and the variable factors are shown. For response data, the information of rheology and antioxidant activity showing the average of three independent experiments and the information data of ATPs is relative to the average of three independent experiments of timepoint day three.

Run	Coded Level			Factors			Response Data		
	*x*_1_	*x*_2_	*x*_3_	Temp (°C)	[jCOL]	[aFUC]	Rheological (G′)	Antiox (µg/mL)	ATPs (RLU)
**1**	−1	−1	−1	−20	3	5	1.13 × 10^4^	3.14 × 10^−2^	7.67 × 10^4^
**2**	−1	−1	0			10	139 × 10^4^	6.51 × 10^−2^	1.90 × 10^5^
**3**	−1	−1	1			15	9.73 × 10^3^	2.45 × 10^−1^	1.35 × 10^5^
**4**	−1	0	−1		5	5	5.73 × 10^5^	8.22 × 10^−3^	5.00 × 10^4^
**5**	−1	0	0			10	6.46 × 10^4^	7.04 × 10^−2^	2.22 × 10^5^
**6**	−1	0	1			15	6.96 × 10^3^	2.36 × 10^−1^	2.61 × 10^5^
**7**	−1	1	−1		7	5	4.67 × 10^4^	2.51 × 10^−2^	9.62 × 10^4^
**8**	−1	1	0			10	2.67 × 10^4^	6.19 × 10^−2^	2.49 × 10^5^
**9**	−1	1	1			15	7.78 × 10^3^	1.66 × 10^−1^	1.79 × 10^5^
**10**	0	−1	−1	−80	3	5	1.84 × 10^4^	2.51 × 10^−2^	7.26 × 10^4^
**11**	0	−1	0			10	2.11 × 10^4^	1.04 × 10^−1^	2.61 × 10^5^
**12**	0	−1	1			15	7.97 × 10^3^	1.70 × 10^−1^	1.75 × 10^5^
**13**	0	0	−1		5	5	6.66 × 10^4^	2.30 × 10^−2^	7.79 × 10^4^
**14**	0	0	0			10	8.04 × 10^4^	2.53 × 10^−1^	2.27 × 10^5^
**15**	0	0	1			15	1.11 × 10^4^	2.69 × 10^−1^	2.30 × 10^5^
**16**	0	1	−1		7	5	5.09 × 10^4^	1.72 × 10^−2^	6.75 × 10^4^
**17**	0	1	0			10	4.10 × 10^4^	3.93 × 10^−2^	2.11 × 10^5^
**18**	0	1	1			15	1.40 × 10^4^	2.55 × 10^−1^	1.93 × 10^5^
**19**	1	−1	−1	−196	3	5	2.46 × 10^3^	1.93 × 10^−2^	5.36 × 10^4^
**20**	1	−1	0			10	1.01 × 10^4^	1.96 × 10^−1^	1.99 × 10^5^
**21**	1	−1	1			15	6.78 × 10^3^	2.81 × 10^−1^	2.89 × 10^5^
**22**	1	0	−1		5	5	1.54 × 10^4^	2.67 × 10^−2^	6.82 × 10^4^
**23**	1	0	0			10	2.53 × 10^4^	2.92 × 10^−2^	3.12 × 10^5^
**24**	1	0	1			15	6.41 × 10^3^	1.93 × 10^−1^	4.38 × 10^5^
**25**	1	1	−1		7	5	1.16 × 10^4^	3.14 × 10^−2^	8.14 × 10^4^
**26**	1	1	0			10	7.91 × 10^3^	9.99 × 10^−2^	2.30 × 10^5^
**27**	1	1	1			15	6.66 × 10^3^	2.73 × 10^−1^	2.27 × 10^5^

**Table 4:** Optimum conditions determined by surface factorial design and the predicted value according to the optimum values.

Response	Factors	Optimum Values	Predicted Value
**G′/Pa**	Temp (°C)	−126	6.47 × 10^4^
	[collagen] mg/mL	5.3	
	[fucoidan] mg/mL	7.7	
**Anti-oxidants (µg/mL)**	Temp (°C)	−54	0.13
	[collagen] mg/mL	3.5	
	[fucoidan] mg/mL	NF	
**ATPs (RLU)**	Temp (°C)	−145	2.56 × 10^5^
	[collagen] mg/mL	5.1	
	[fucoidan] mg/mL	12.5	

NF: not found or extrapolated.

**Table 5:** Analysis of variance (ANOVA) for the fitted quadratic polynomial model for optimization of cryogel manufacturing.

Variable of G′/Pa					
Factor	Sum of Squares (SS)	df	Mean Square (MS)	*F*-Value	*p*-Value
**(1) [fucoidan] (L)**	2.29 × 10^9^	1	2.29 × 10^9^	11.766	0.002
**[fucoidan] (Q)**	9.28 × 10^8^	1	9.28 × 10^8^	4.757	0.041
**(2) [collagen] (L)**	6.92 × 10^8^	1	6.92 × 10^8^	3.547	0.074
**[collagen] (Q)**	2.31 × 10^9^	1	2.31 × 10^9^	11.855	0.002
**(3) Temp (°C) (L)**	1.29 × 10^9^	1	1.29 × 10^9^	6.616	0.018
**Temp (°C) (Q)**	2.00 × 10^9^	1	2.00 × 10^9^	10.246	0.004
**Error**	3.90 × 10^9^	20	1.95 × 10^8^		
**Total SS**	1.29 × 10^10^	26			
R^2^ = 0.69; adj R^2^ = 0.61; MS = 195 × 10^3^					
**Variable of Anti-oxidant (µg/mL)**					
**Factor**	**Sum of squares (SS)**	**df**	**Mean square (MS)**	***F*-value**	***p*-value**
**(1) Temp (°C) (L)**	0.003	1	0.003	1.170	0.292
**Temp (°C) (Q)**	0.000	1	0.000	0.203	0.656
**(2) [collagen] (L)**	0.001	1	0.001	0.560	0.462
**[collagen] (Q)**	0.000	1	0.000	0.085	0.773
**(3) [fucoidan] (L)**	0.196	1	0.196	71.419	0.000
**[fucoidan] (Q)**	0.003	1	0.003	1.412	0.248
**Error**	0.054	20	0.002		
**Total SS**	0.261	26			
R^2^ = 0.78; adj R^2^ = 0.72; MS = 0.00					
**Variable of ATPs (RLU)**					
**Factor**	**Sum of squares (SS)**	**df**	**Mean square (MS)**	***F*-value**	***p*-value**
**(1) Temp (°C) (L)**	1.07 × 10^10^	1	1.07 × 10^10^	4.158	0.054
**Temp (°C) (Q)**	3.90 × 10^9^	1	3.90 × 10^9^	1.515	0.232
**(2) [collagen] (L)**	3.68 × 10^8^	1	3.68 × 10^8^	0.143	0.709
**[collagen] (Q)**	1.13 × 10^10^	1	1.13 × 10^10^	4.422	0.048
**(3) [fucoidan] (L)**	1.22 × 10^11^	1	1.22 × 10^11^	47.455	0.000
**[fucoidan] (Q)**	3.79 × 10^10^	1	3.79 × 10^10^	14.715	0.001
**Error**	5.15 × 10^10^	20	2.57 × 10^9^		
**Total SS**	2.36 × 10^11^	26			
R^2^ = 0.78; adj R^2^ = 0.71; MS = 257 × 10^4^					

R^2^: regression goodness of fit. df: degrees of freedom. L: linear. Q: quadratic.

## Data Availability

Not applicable.
